# Real‐World Effectiveness of Mepolizumab in Patients With Chronic Rhinosinusitis With Nasal Polyps: Findings From the European CRS Outcome Registry (CHRINOSOR)

**DOI:** 10.1002/clt2.70153

**Published:** 2026-01-31

**Authors:** Isam Alobid, Sven F. Seys, Joost de Kinderen, Valérie Hox, Carlo Cavaliere, Alexandros Andrianakis, Sven Schneider, Martin Wagenmann, Martin Bruch, Adriana Izquierdo‐Domínguez, Xavier Gonzalez‐Compta, Laura Pardo Muñoz, Laura Van Gerven, Peter W. Hellings, Geoffrey Mortuaire, Martin Burian, Mireia Golet‐Fors, Mathilde Moyaert, Peter‐Valentin Tomazic, Giulia Bettio, Marco de Vincentiis, Zuzana Diamant, Julia Eckl‐Dorna, Gert Mariën, Simonetta Masieri, Christina Morgenstern, Kathrin Scheckenbach, Aldine Tu, Camilo Rodriguez van Strahlen, Claus Bachert, CHRINOSOR consortium

**Affiliations:** ^1^ Otorhinolaryngology Department Rhinology Unit & Smell Clinic and Skull Base Surgery Unit Hospital Clinic Barcelona Universitat de Barcelona IDIBAPS CIBERES Barcelona Catalonia Spain; ^2^ Galenus Health (part of Cascador Health Group) Tielrode Belgium; ^3^ Service d'Otorhinolaryngologie Cliniques Universitaires Saint‐Luc Brussels Belgium; ^4^ Department of Life Sciences Health and Health Professions Link Campus University Rome Italy; ^5^ Department of General ORL, Head and Neck Surgery Medical University of Graz Graz Austria; ^6^ Department of Otorhinolaryngology, Head and Neck Surgery Vienna General Hospital (AKH) Medical University of Vienna Vienna Austria; ^7^ Department of Otorhinolaryngology Universitätsklinikum Düsseldorf (UKD) Dusseldorf Germany; ^8^ Department of Otorhinolaryngology Ordensklinikum Linz Linz Austria; ^9^ Allergology Department Hospital Universitario de Terrassa Barcelona Spain; ^10^ Rhinology Unit, Otorhinolaryngology Department, Hospital Universitari de Bellvitge Universitat de Barcelona, IDIBELL Barcelona Catalonia Spain; ^11^ Otorhinolaryngology Department Hospital Universitari Germans Trias i Pujol Barcelona Spain; ^12^ Department of Otorhinolaryngology‐Head and Neck Surgery UZ Leuven Leuven Belgium; ^13^ Department of Microbiology, Immunology & Transplantation Allergy and Clinical Immunology Research Group KU Leuven Leuven Belgium; ^14^ Department of Neurosciences, Experimental Otorhinolaryngology Rhinology Research KU Leuven Leuven Belgium; ^15^ Otorhinolaryngology‐Head and Neck Department Huriez Hospital Centre Hospitalier Universitaire (CHU) Lille Lille France; ^16^ Department of Respiratory Medicine First Faculty of Medicine Charles University and Thomayer Hospital Prague Czech Republic; ^17^ Department of Clin Pharm & Pharmacol University Groningen University Medical Center Groningen Groningen the Netherlands; ^18^ Department of Oral and Maxillofacial Sciences Sapienza University Rome Italy; ^19^ Clinic for ENT diseases and Head and Neck Surgery University Clinic Münster Münster Germany; ^20^ Department of Otorhinolaryngology First Affiliated Hospital Sun Yat‐Sen University International Airway Research Center Guangzhou China

**Keywords:** asthma, biologic, CRSwNP, mepolizumab, real world evidence, registry

## Abstract

**Background:**

The SYNAPSE phase 3 study demonstrated that mepolizumab significantly improves nasal polyp score (NPS), quality of life and symptom severity in patients with chronic rhinosinusitis with nasal polyps (CRSwNP).

**Objective:**

We aimed to evaluate mepolizumab effectiveness in a real‐world cohort from 12 tertiary centers in 6 European countries.

**Methodology:**

A retrospective analysis was conducted in 110 CRSwNP patients (comorbid asthma: 86.4%). CRS‐related outcomes were analyzed at baseline, 24 and 52 weeks of mepolizumab. Treatment response was evaluated based on EUFOREA 2021 criteria.

**Results:**

Significant improvements in NPS, Sinonasal Outcome Test‐22 (SNOT‐22), and visual analog scale (VAS) symptom scores were observed at 24 and 52 weeks compared to baseline. Further improvement between weeks 24 and 52 was observed for NPS and SNOT‐22. Asthma Control Test (ACT) also improved significantly by week 24 (ACT score ≥ 20: 64.5%). At least one response criterion (change in SNOT‐22 ≥ 8.9, NPS ≥ 1, VAS total sinus symptoms ≥ 20, VAS nasal blockage ≥ 20, VAS loss of smell ≥ 20) was met by 85.6% and 78.7% of patients at 24 and 52 weeks, respectively. A more stringent composite response (SNOT‐22 < 30, NPS < 4, VAS total sinus symptoms < 50, and VAS nasal blockage < 50) was achieved in 18.3% and 44.6% of patients at 24 and 52 weeks, respectively.

**Conclusion:**

Mepolizumab demonstrated clinically meaningful benefits in a real‐world CRSwNP population, with nearly half of patients achieving a beneficial composite treatment response by week 52. Notably, progressive improvements between weeks 24 and 52 underscore the value of prolonged therapy and the importance of evaluating treatment response at one year.

## 1 Introduction

Chronic rhinosinusitis (CRS) affects approximately 11% of the European population [1]. Approximately 20%–30% of CRS patients are diagnosed with nasal polyps (CRSwNP). CRSwNP is characterized by a predominantly type 2 inflammatory response mediated by interleukins (IL‐)4, 5 and 13, along with tissue eosinophilic infiltration, blood eosinophilia and increased total serum Immunoglobulin E (IgE) in Western countries, whereas nasal polyps from Asian patients often have more a type 1 or 3 endotype [2, 3]. Common type 2 comorbidities include asthma and non‐steroidal anti‐inflammatory drug exacerbated respiratory disease (N‐ERD), which are more prevalent in patients with CRSwNP than CRSsNP [4, 5, 6]. Presence of type 2 inflammation has been linked with more severe disease [7]. According to EPOS2020, symptom severity and impact on quality of life (QoL) can be assessed by visual analog scale (VAS) and the Sinonasal Outcome Test‐22 (SNOT‐22) [8]. VAS total sinonasal symptoms (TSS) showed to correlate well with SNOT‐22 and can be used as a simple tool to assess the CRS burden [9].

Biologic therapies including dupilumab, mepolizumab and omalizumab, target the type 2 inflammatory cascade and showed beneficial outcomes on various clinical CRS outcomes, such as nasal polyps score (NPS), SNOT‐22, nasal blockage and loss of smell [10, 11, 12]. This has led to their market authorization for the treatment of uncontrolled severe CRSwNP in many regions globally. The goal of treatment with biologics is to achieve optimal disease control and reduce impact on QoL [13]. Preliminary evidence also confirmed their potential as inducers of remission in some patients [14].

Specifically to mepolizumab, the phase 3 SYNAPSE study showed a significant reduction of nasal obstruction VAS scores (−3.14; 95% CI: −4.09 to −2.18) and NPS (−0.73; 95% CI: −1.11 to −0.34) [11]. Secondary outcomes demonstrated a reduction in SNOT‐22 scores, overall symptoms VAS, and improved sense of smell. A recent phase 3 study conducted in Japan, China and Russia confirmed mepolizumab efficacy on nasal obstruction VAS as primary outcome but could not demonstrate a significant effect on NPS at 52 weeks [15]. Interestingly, in another study, mepolizumab also showed reduced need for sinus surgery compared to placebo at 25 weeks of treatment [16].

While randomized clinical trials (RCT) provide robust efficacy data on regulatory recommended outcomes, real‐world studies add complementary insights validating these clinical outcomes in real‐world CRSwNP cohorts. Real‐world studies on mepolizumab effectiveness with CRSwNP as primary indication so far were reported in national cohorts only with patient numbers not allowing for subgroup stratification or follow up until 24 weeks [17, 18, 19, 20]. Therefore, larger studies providing real‐world evidence from multiple countries allowing for evaluation of the impact of comorbidities and other real‐world variables are needed. The CHronic RhINOSinusitis Outcome Registry (CHRINOSOR) was conceived to address these research needs in the European context [21]. In the current study, we evaluated mepolizumab effectiveness in 110 CRSwNP patients from 6 European countries up to 52 weeks.

## 2 Methods

### 2.1 Study Design and Population

This was a retrospective, multicenter study conducted in 12 tertiary care centers from 6 European countries (see list of participating centers in the Online Repository). Study methods are in line with the previously published dupilumab effectiveness analysis in the CHRINOSOR cohort [22]. Data from patients treated with mepolizumab between December 2019 and December 2024 were analyzed. Pseudonymized data were collected from the (electronic) health records at initiation of mepolizumab treatment (baseline), after 24 (± 8) weeks and 52 (± 8) weeks of treatment using a standardized data collection protocol across participating centers to minimize heterogeneity. The study was approved by the local institutional review boards (France: not required as long as clear information and consent has been reported) and registered at clinicaltrials.gov (NCT04670172).

### 2.2 Inclusion and Exclusion Criteria

In total, 110 patients were treated with mepolizumab (100 mg subcutaneous every 4 weeks) for uncontrolled severe CRSwNP in the real‐world setting. Patients eligible for the treatment according to national indications (Table S1) were included in the study if they had at least one data point for nasal polyp score (NPS), SNOT‐22, or VAS at 24 and/or 52 weeks. More detailed information can be found in the Online Repository.

### 2.3 Evaluation of Treatment Response

EUFOREA 2021 criteria were applied to assess the response to mepolizumab [23]. Since nasal congestion score and loss of smell score are not routinely applied, VAS nasal blockage and VAS loss of smell were analyzed as part of a standardized evaluation of symptoms by VAS as described above. At 24 weeks, patients were considered responsive to biologics if at least one score had improved: VAS total sinus symptoms (change compared to baseline ≥ 20 mm), VAS loss of smell (change compared to baseline ≥ 20 mm), VAS nasal blockage (change compared to baseline ≥ 20 mm), NPS (change compared to baseline ≥ 1), SNOT‐22 (change compared to baseline ≥ 8.9) [23]. At 52 weeks, patients were considered to have a beneficial composite response if all of the following criteria were fulfilled: VAS nasal obstruction (< 50 mm), VAS total sinus symptoms (< 50 mm), NPS (< 4), SNOT‐22 score (< 30) [23]. Additionally, the need for a course of SCS during biologic treatment was assessed.

### 2.4 Statistics

Sample size calculations were performed for NPS and SNOT‐22 score based on historical data of the previous mepolizumab phase 3 trial [11]. Details can be found in the Online Repository. Within‐group comparison of the outcome parameters was performed by mixed effects model (restricted maximum likelihood without data imputation for missing values) and Dunnett's, Sidak's or Tukey's multiple comparison test. A normal distribution of the residuals was observed. Geisser‐Greenhouse correction was applied. Missing data were reported for each outcome parameter in the figure legends. Proportions of patients were compared by Chi^2^ test. Data were presented as Tukey box‐and‐whisker plots (median with interquartile range) without data transformation. Statistical analysis was performed using GraphPad Prism 9 software (Boston, US). A *p*‐value of less than 0.05 was considered statistically significant.

## 3 Results

### 3.1 Patient Characteristics

Included patients (*n* = 110) presented with a variable history of number of ESS procedures (85.0% with prior ESS) and number of SCS courses in the past year (70.4% with prior SCS courses). Prior biologic therapy for CRSwNP and/or asthma was reported in 11.8% (13 in total) of patients. Regarding comorbidities, 86.4% of patients had a history of comorbid asthma, 36.2% a clinical diagnosis of N‐ERD and 58.3% had any type of allergic disease. The majority of patients (87.8%) showed signs of systemic type 2 inflammation (defined by eosinophils/mm^3^ ≥ 150 and/or serum total IgE ≥ 100 IU/mL as in EPOS/EUFOREA 2023 [24]). 7.4% of patients had 150–299 eosinophils/mm^3^, 74.7% ≥ 300 eosinophils/mm^3^ and: 55.3% serum total IgE ≥ 100 IU/mL. Baseline patient characteristics were summarized in Table 1.

**TABLE 1:** Patient characteristics.

	Mepolizumab (*n* = 110)
Age, mean +/– SD	57.0 +/– 12.6
Male − female (%)	55.0 – 45.0
BMI, mean +/− SD	25.4 +/− 3.9
Smoking status (never ‐ ex − current), %	73.4 – 21.9 – 4.7
Allergy, %	58.3
Asthma, %	86.4
N‐ERD, %	36.2
# ESS procedures (0 – 1 – 2 – 3 – > 3), %	15.0 – 33.6 – 21.5 – 14.0 – 15.9
SCS courses past year (0 – 1 – 2 – 3 – > 3), %	34.0 – 18.1 – 22.3 – 5.3 – 20.2
Prior biologics, %	11.8
NPS (0 to 2 – 3 to 4 – 5 to 6 – 7 to 8), %	14.3 – 33.3 – 41.9 – 10.5
Lund‐Mackay score, median [IQR]	17.5 [14.0–20.3]
VAS total sinus symptoms, median [IQR]	51.0 [30.0–80.0]
VAS loss of smell, median [IQR]	97.0 [77.0–100.0]
VAS nasal blockage, median [IQR]	70.0 [57.5–90.0]
SNOT‐22, median [IQR]	56.0 [42.0–69.3]
ACT, median [IQR]	17.0 [13.8–22.0]
Blood eosinophils, median [IQR]	500.0 [275.0–860.0]
Serum total IgE, median [IQR]	110.0 [50.5–312.0]

### 3.2 Effectiveness of Mepolizumab on NPS and SNOT‐22

**FIGURE 1:**
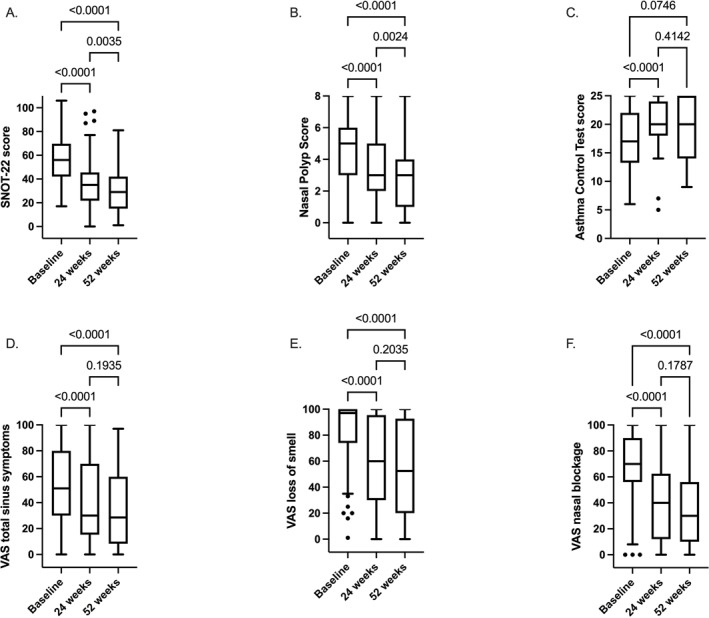
Mepolizumab treatment effect on NPS, SNOT‐22, ACT, VAS TSS, VAS LoS and VAS NB. (A), Patient numbers of SNOT‐22: 96, 94 and 71. (B), Patient numbers of NPS: 105, 94 and 84. (C), Patient numbers of ACT: 44, 31 and 23. (D), Patient numbers of VAS total sinus symptoms: 81, 88 and 68. (E)Patient numbers of VAS LoS: 83, 93 and 74. (F), Patient numbers of VAS nasal blockage: 75, 81 and 63. Data are presented as Tukey box‐and‐whisker plots. Within‐group comparison was performed by mixed effects model and Dunnett multiple testing comparison.

NPS significantly improved at both 24 weeks (3.0 [2.0–5.0]) and 52 (3.0 [1.0–4.0]) compared to baseline (5.0 [3.0–6.0]; *p* < 0.0001 for both; Figure 1A). At 52 weeks, a further decrease in NPS compared to 24 weeks was demonstrated (*p* = 0.002; Figure 1A), with an improvement of NPS ≥ 1 in 61.3% and 59.8% of patients at 24 and 52 weeks, respectively (Table 2).

**TABLE 2:** Mepolizumab effectiveness based on EUFOREA 2021 criteria.

	Applied criteria	% responders at 24 weeks	% responders at 52 weeks	% change 52 versus 24 weeks
Responder defined as one of these five criteria to be met	**Diff SNOT‐22** ≥ **8.9**	69 out of 92 = **75.0%**	56 out of 69 = 81.2%	+6.2%
	**Diff NPS 1 or** ≥ **1**	57 out of 93 = **61.3%**	49 out of 82 = 59.8%	−1.5%
	**Diff VAS TSS** ≥ **20**	28 out of 81 = **34.6%**	27 out of 63 = 42.9%	+8.3%
	**Diff VAS NB** ≥ **20**	47 out of 75 = **62.7%**	39 out of 58 = 67.2%	+4.6%
	**Diff VAS LoS** ≥ **20**	42 out of 83 = **50.6%**	34 out of 64 = 53.1%	+2.5%
At least one of these 5 criteria met		89 out of 104 = **85.6%**	70 out of 89 = 78.7%	−6.9%
Responder defined as all of these four criteria to be met	**SNOT‐22 < 30**	36 out of 94 = 38.3%	39 out of 71 = **54.9%**	+16.6%
	**NPS < 4**	52 out of 94 = 55.3%	55 Out of 84 = **65.5%**	+10.2%
	**VAS TSS < 50**	54 out of 88 = 61.4%	41 out of 68 = **60.3%**	−1.1%
	**VAS NB < 50**	42 out of 81 = 51.9%	42 out of 63 = **66.7%**	+14.8%
Additional criterion	**VAS LoS** **< 50**	34 out of 93 = 36.6%	34 out of 74 = 45.9%	+9.4%
All these 4 criteria met		13 out of 71 = 18.3%	25 out of 56 = **44.6%**	+26.3%

*Note:* Percentages in bold indicate the applicable EUFOREA 2021 criteria at that time point.

Also, SNOT‐22 scores significantly improved at both 24 weeks (median [Inter Quartile Range, IQR]: 35.0 [22.0–45.0]) and 52 (29.0 [15.0–41.5]) of mepolizumab treatment compared baseline (56.0 [42.0–69.3]; *p* < 0.0001 for both; Figure 1B). Similarly, a further decrease in SNOT‐22 scores at 52 weeks compared to 24 weeks was found (*p* = 0.004; Figure 1B). A clinically relevant improvement in SNOT‐22 (≥ 8.9) was reached in 75.0% and 81.2% of patients at 24 and 52 weeks, respectively (Table 2).

### 3.3 Effectiveness of Mepolizumab on Symptom Scores

A significant decrease in VAS TSS was observed at both 24 (30.0 mm [15.8–70.0]) and 52 weeks (28.5 mm [8.8–60.0]) of treatment compared to baseline (51.0 mm [30.0–80.0]; *p* < 0.0001 for both; Figure 1D). Also, VAS LoS as well as VAS NB significantly improved at 24 (60.0 mm [30.0–95.0] and 40.0 mm [13.0–60.0]) and 52 weeks (52.5 mm [20.8–92.0] and 30.0 mm [11.5–55.5]) compared to baseline (97.0 mm [77.0–100.0] and 70.0 mm [57.5–90.0]; *p* < 0.0001 for both; Figure 1E, F). No further decrease at 52 weeks compared to 24 weeks was observed for TSS ((*p* = 0.19), LoS (*p* = 0.20), NB (*p* = 0.18); Figure 1D–F).

### 3.4 Analysis of Mepolizumab Responders at 24 and 52 weeks

At 24 weeks, 85.6% of patients fulfilled at least 1 criterion to qualify as responder, which slightly decreased to 78.7% of patients at 52 weeks (Table 2). 18.3% of patients (from those with all 4 variables available) met all 4 qualifying criteria for responders at 24 weeks compared to 44.6% at 52 weeks and 0.0% at baseline (Table 2).

### 3.5 Effectiveness of Mepolizumab on Asthma Control

In patients with comorbid asthma, the ACT score significantly improved at 24 weeks (20.0 [18.5–24.0]), but not at 52 weeks (20.0 [14.5–24.5]) of treatment compared to baseline (17.0 [13.8–22.0]; *p* < 0.0001 and *p* = 0.07; Figure 1C). The proportion of patients with an ACT ≥ 20 increased from 34.1% at baseline to 64.5% and 56.5% at 24 and 52 weeks respectively (*p* = 0.02 and *p* = 0.12).

### 3.6 Need for SCS Use During Mepolizumab Treatment

The proportion of patients requiring a course of SCS during mepolizumab treatment reduced from 70.4% at baseline to 31.1% and 30.8% at 24 and 52 weeks respectively (*p* < 0.0001 and *p* < 0.0001).

### 3.7 Effectiveness of Mepolizumab in Patients With Comorbid Asthma, N‐ERD, or Allergy

**FIGURE 2:**
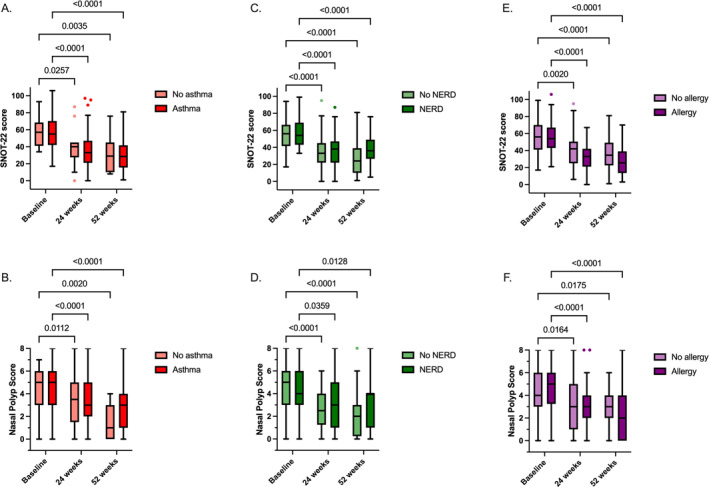
Mepolizumab treatment effect on NPS and SNOT‐22, stratified by asthma, N‐ERD and allergy. (A), Patient numbers of SNOT‐22: no asthma: 14, 13 and 11; asthma: 82, 81 and 60. (B), Patient numbers of NPS: no asthma: 13, 14 and 11; asthma: 92, 80 and 73. (C), Patient numbers of SNOT‐22: no N‐ERD: 49, 47 and 39; N‐ERD: 31, 32 and 25. (D), Patient numbers of NPS: no N‐ERD: 56, 48 and 52; N‐ERD: 33, 31 and 23. (E), Patient numbers of SNOT‐22: no allergy: 31, 29 and 22; allergy: 53, 52 and 44. (F), Patient numbers of NPS: no allergy: 39, 33 and 31; allergy: 52, 48 and 46. Data are presented as Tukey box‐and‐whisker plots. Within‐group comparison was performed by mixed effects model and Dunnett multiple testing comparison.

Mepolizumab effectiveness was analyzed in patients stratified by the presence of comorbid asthma, N‐ERD or allergy. Mepolizumab showed significant improvements in NPS and SNOT‐22 in each of the subgroups, both at 24 and 52 weeks compared to baseline (Figure 2A–F). No significant differences in the change to baseline at 24 and 52 weeks were observed between patients with or without any of the listed comorbidities, but patients with N‐ERD and allergy showed a greater improvement in NPS at 52 weeks compared to patients without presence of these comorbidities (*p* = 0.01 and *p* = 0.002; Figures S1–S3).

### 3.8 Effect of Mepolizumab on Type 2 Inflammatory Markers At 24 and 52 weeks

**FIGURE 3:**
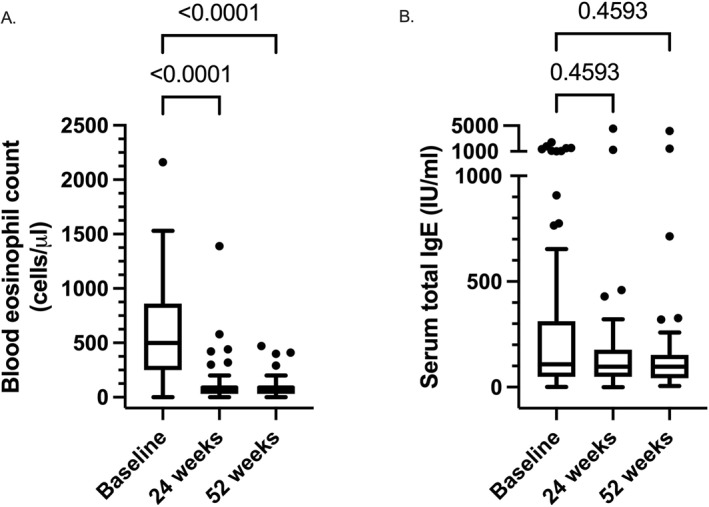
Mepolizumab treatment effect on NPS and SNOT‐22, stratified by baseline BEC. A, Patient numbers of BEC: 95, 67 and 53. B, Patient numbers of serum total IgE: 84, 51 and 45. Data are presented as Tukey box‐and‐whisker plots. Within‐group comparison was performed by mixed effects model and Dunnett multiple testing comparison.

Mepolizumab significantly reduced BEC at 24 (90.0 cells/mm^3^ [30.0–100.0]) and 52 (90.0 cells/mm^3^ [30.0–100.0]) weeks compared to baseline (500.0 cells/mm^3^ [275.0–860.0]; *p* < 0.0001 for both; Figure 3A). Serum total IgE levels were not significantly altered after 24 (98.0 IU/mL [49.1–173.3] and 52 97.5 IU/mL [48.3–152.3]) weeks of treatment compared to baseline (108 IU/mL [50.5–291.0]; *p* = 0.46 for both; Figure 3B).

### 3.9 Effectiveness of Mepolizumab Based On Baseline BEC

**FIGURE 4:**
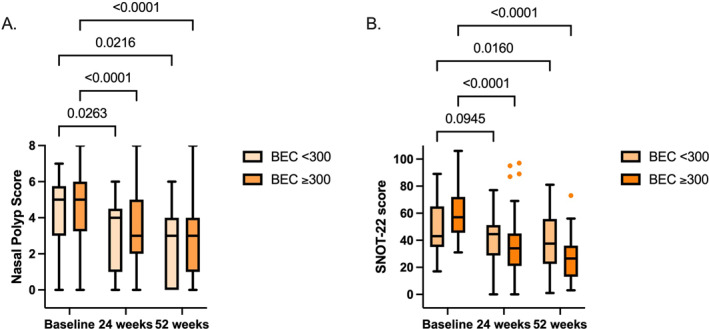
Mepolizumab treatment effect on BEC and serum total IgE. (A), Patient numbers of NPS: BEC < 300: 24, 21 and 19; BEC ≥ 300: 68, 63 and 54. (B), Patient numbers of SNOT‐22: BEC < 300: 23, 22 and 18; BEC ≥ 300: 61, 59 and 42. Data are presented as Tukey box‐and‐whisker plots. Within‐group comparison was performed by mixed effects model and Dunnett multiple testing comparison.

Patients with BEC ≥ 300 cells/mm^3^ showed a significant improvement in NPS and SNOT‐22, both at 24 and 52 weeks (*p* < 0.0001 for both; Figure 4A,B). Patients with BEC < 300 cells/mm^3^ also showed significant improvements in NPS at 24 (*p* = 0.03) and 52 (*p* = 0.02) weeks and in SNOT‐22 at 52 weeks (*p* = 0.02) but not at 24 weeks (*p* = 0.09; Figure 4A,B). Patients with BEC ≥ 300 cells/mm^3^ showed a significantly larger improvement (change to baseline) in SNOT‐22 both at 24 and 52 weeks compared to patients with BEC < 300 cells/mm^3^ (*p* = 0.004 and *p* = 0.0002; Figure S4), whereas no differences in change to baseline were observed for NPS at 24 and 52 weeks.

### 3.10 Analysis of Prior Biologics

Out of the 13 patients with prior biologic use for CRSwNP and/or asthma (7 dupilumab, 4 omalizumab, and 2 reslizumab), 9 patients (69.2%) switched to mepolizumab because of insufficient response, whilst 1 patient (7.7%) switched due to side effects (arthritis) and for 3 patients (23.1%) the reason was unknown. At 24 weeks, 84.6% of patients with prior biologic use fulfilled at least 1 criterion to qualify as responder, which decreased to 60.0% of patients at 52 weeks. Compared to the 97 patients who were not on a prior biologic, these proportions were similar at 24 weeks (85.7%; *p* = 0.92) or at 52 weeks (81.0%; *p* = 0.13). At 52 weeks, 22.2% of patients with prior biologic use met all 4 more stringent criteria for responders, compared to 48.9% of patients not on prior biologic therapy (*p* = 0.14; Table S2).

### 3.11 Analysis of Mepolizumab Discontinuation

**FIGURE 5:**
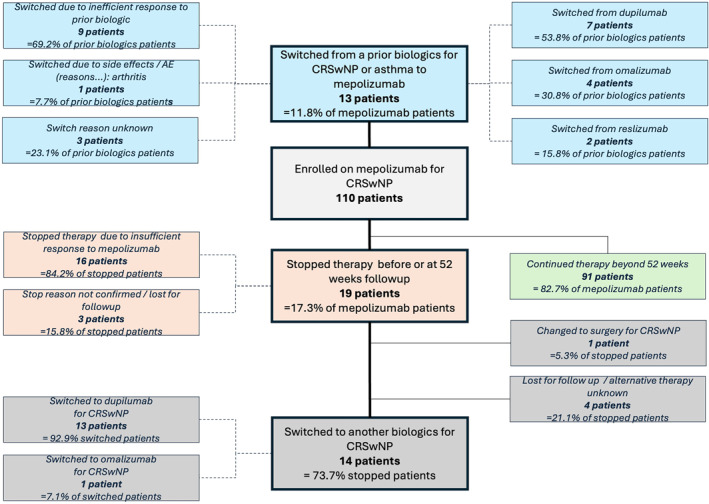
Prior biologic use, discontinuation of mepolizumab treatment and switches.

From the 110 included patients, 19 patients (17.3%) stopped mepolizumab treatment before or at 52 weeks, of whom 16 (84.2%) due to inefficient response, while 3 (15.8%) were lost to follow‐up or their reason for discontinuation was unknown. Of those stopped patients, 26.4% (*n* = 5) switched to non‐biological therapy (ESS or SCS) or were lost to follow‐up, whilst 73.7% (*n* = 14) patients switched from mepolizumab to another biologic: 92.9% (*n* = 13) switched to dupilumab and 7.1% (*n* = 1) to omalizumab. Mepolizumab discontinuation and switches to other biologics are summarized in Figure 5.

### 3.12 Analysis of Mepolizumab Responders in Discontinued Therapy Patients

At 24 weeks, 73.7% (14/19) of patients who discontinued mepolizumab in the 52‐week study period fulfilled at least 1 criterion to qualify as responder, compared to 88.2% (75/85) of patients who continued mepolizumab at least until 52 weeks (*p* = 0.10). Of those who discontinued, 15.4% of patients met all 4 more stringent qualifying criteria at 24 weeks, compared 19.0% of the patients who did not discontinue (*p* = 0.76). Due to discontinuations, the number of patients with outcomes at 52 weeks was too low to meaningfully assess response at that timepoint (Table S2).

## 4 Discussion

We here report on the first international multicenter study evaluating mepolizumab effectiveness for CRSwNP in the real‐world setting. Our results confirmed the beneficial effects of mepolizumab for uncontrolled severe CRSwNP with almost half of patients showing a beneficial composite response at 52 weeks of treatment as defined by EUFOREA 2021 [23]. Importantly, an additional improvement in NPS and SNOT‐22 score at 52 weeks compared to 24 weeks was demonstrated, with the maximum effects on LoS and NB already reached at 24 weeks. Stratification of patients by the presence of comorbidities furthermore showed effectiveness in each of the subgroups at both timepoints.

Uncontrolled severe CRSwNP patients often present with comorbid asthma or N‐ERD [25]. In our cohort, asthma was present in 86.4% of patients being slightly higher than reported in most other real‐world studies with mepolizumab (62.1%–92.6%). The prevalence of N‐ERD was 36.2%, which aligns with existing literature [26, 27]. In comparison with SYNAPSE, we report higher numbers of both comorbid asthma (86.4% vs. 68.0%) and N‐ERD (36.2% vs. 21.8%, Table S3). Effectiveness of mepolizumab was maintained in patients with or without asthma or N‐ERD, with no differences for SNOT‐22 and NPS between the subgroups. This is in line with other studies that showed similar effectiveness of mepolizumab considering the patient's presence of asthma or atopy [28].

Other real‐world mepolizumab studies evaluated the effect on sinonasal outcomes in severe asthma [27, 29]. Despite the beneficial effects of different type 2 directed biologics on both CRSwNP and asthma, their relative contribution to blocking disease activity in these diseases still needs further investigation [30]. Related to this, a recent study in severe asthma patients treated with mepolizumab showed that patients who had comorbid CRSwNP showed higher rates of super‐responders compared to patients without CRSwNP [31]. Patients with co‐morbid disease had a higher combined bone marrow IL‐5 drive, as reflected by higher blood eosinophils in those with co‐morbid disease compared to those with single organ disease. This could explain why clinicians may have favored mepolizumab as a treatment option in clinical practice for those with co‐morbid asthma and higher BEC (≥ 300 cells/mm^3^). Indeed, we found a high proportion of patients with comorbid asthma and/or N‐ERD as well as high median baseline BEC in our real‐world cohort of patients treated with mepolizumab.

Comparing our results with other real‐world data on mepolizumab treatment for the primary indication of CRSwNP showed similar effects on NPS, SNOT‐22 and symptom scores [17, 20, 32]. Dominguez‐Sosa et al. showed an even larger effect on SNOT‐22 and symptoms already at 24 weeks, likely due to the very high number of asthmatics (89%) in their cohort [26].

As shown in the literature mepolizumab significantly reduces BEC [15, 16, 17, 18], and this was confirmed in our cohort. With respect to effectiveness, mepolizumab showed a more pronounced effect for SNOT‐22 in patients with a baseline BEC ≥ 300 cells/mm^3^ as compared to patients with baseline BEC < 300 cells/mm^3^, suggesting that mepolizumab is more effective in patients with a higher BEC in the real‐world setting. This is expected given that mepolizumab selectively blocks IL‐5 which translates into an inhibition of eosinophil maturation, proliferation and activation [33]. Interestingly, multiple immune cells may in fact be a target, as shown in mepolizumab treated N‐ERD patients, and furthermore IL‐5 has direct relevance to structural cell activation that would be of relevance to tissue remodeling and nasal polyp growth [34, 35]. The increased effect in patients with baseline BEC ≥ 300 cells/mm^3^ was however not observed for NPS.

A median reduction in NPS of 2 points was observed in our study, which indicated an effect that is double the result that was observed in SYNAPSE. The percentage of patients with an NPS reduction of ≥ 1 and ≥ 2 at 52 weeks was 59.8% and 47.6%, which is also larger improvement than reported in SYNAPSE (50.6% and 36.0%, respectively). Remarkably, a recent phase 3 study from Japan, China and Russia was not able to demonstrate a significant effect on NPS at 52 weeks [15]. This may be due to regional differences and the fact that it has been shown before that a lower proportion of Asian CRSwNP patients have underlying type 2 inflammation. As described in our study, NPS decreased over the time with a further significant improvement from 24 to 52 weeks of treatment, indicating that a time frame of 12 months may be adequate to evaluate relevant CRS outcomes. This seems slower than what has been observed with dupilumab in CRSwNP patients [10, 22]. On the other hand, discontinuation of dupilumab led to a relatively fast recurrence of nasal polyps, whereas this process seemed slower or almost absent after mepolizumab discontinuation, suggestive of a basic effect on the underlying disease process with IL‐5 inhibition by mepolizumab [10, 36]. The percentage of patients requiring SCS during mepolizumab 52‐week treatment was 30.8% (compared to SCS in the year before baseline of 70.4%), compared to 25.4% at the 52‐week follow‐up in SYNAPSE (51.5% at baseline) [11]. No such RWE data has been reported before except for a smaller study by Orlando et al., who showed a reduced need for OCS in 92.6% (25/27 patients) [28].

In our cohort, 17.3% of patients discontinued mepolizumab treatment before or at 52 weeks, mostly due to insufficient response. Viskens et al. found already 17% of patients that stopped mepolizumab treatment at 24 weeks of treatment, whereas Barhold et al. showed a discontinuation rate of 28.1% (27/96) in patients with a mean follow up of 10.4 months (range 6–30 months) [20, 37].

Despite the advent of biologics allowing effective treatment of CRSwNP, ESS remains an important treatment strategy for CRSwNP patients with uncontrolled disease. According to EPOS 2020 [8] and in most countries, prior ESS is considered a prerequisite before engaging on biologics. According to our findings, SNOT‐22 significantly improved in patients with or without prior ESS at 52 weeks. However, patients without prior ESS did not show significant SNOT‐22 improvement at 24 weeks nor a significant improvement in NPS at both 24 or 52 weeks. While ESS procedures should include opening all diseased sinuses, it is difficult to assess the extent of prior surgery performed in CRSwNP patients, which hampers data interpretation and comparison between studies. Comparison of effectiveness of biologics with ESS or ESS in combination with biologics has not been studied adequately yet to draw firm conclusions. In fact, this is an area of research that is currently being explored. Pelletier et al. showed that 10 weeks perioperative treatment with dupilumab was associated with improved olfactory outcomes at 52 weeks [38]. Another study showed that six months of mepolizumab treatment in addition to ESS results in improvements in SNOT‐22 and NPS as compared to mepolizumab only [39].

In the CHRINOSOR real‐world cohort, 17.3% of patients discontinued mepolizumab within 52 weeks, primarily due to insufficient response and 73.7% of those who stopped mepolizumab treatment (12.7% of total cohort of 110 patients) switched to another biologic, with the vast majority switching to dupilumab. A similar observation was reported in the Canadian multicenter study by Dorling et al. (2025), where 16.0% of patients (36 out of 225) switched biologics during treatment [40], with the most frequent switch also from mepolizumab to dupilumab, and with inadequate control of CRSwNP symptoms cited as the reason in 66.7% of these cases. With regard to patients who received another biologic prior to mepolizumab treatment, no differential response was observed compared to biologic naïve patients. One other study also investigated switched mepolizumab patients and found that these patients showed worse outcomes as compared to biologic naïve patients [37].

While real‐world data support generalizability of RCTs, limitations include retrospective data collection, gaps in follow‐up data availability and the fact that patients were recruited at academic centers only. Also the lack of data on olfactory function tests in this real‐world setting is a limitation. Additionally, while biomarkers such as BEC are promising, algorithms using composite biomarkers identifying patients with a beneficial treatment response remain underdeveloped. Future research should explore head‐to‐head trials, biomarker research and cost‐effectiveness analysis, particularly as biologic use expands.

## 5 Conclusion

Collectively, mepolizumab showed clinically meaningful responses in over 80% of CRSwNP, irrespective of type 2 comorbidities from 24 weeks up to at least 52 weeks. Beneficial composite treatment responses, defined by more stringent criteria, were found in nearly half of mepolizumab treated patients. Given further decrease in NP size and increase in QoL was observed between 24 and 52 weeks, prolonged treatment up to 52 weeks should be considered. However, this should also be evaluated in the context of the dominant symptom reported by the patient and the level of disease control achieved for each individual patient.

## Author Contributions

**Isam Alobid:** conceptualization, investigation, writing – original draft, writing – review and editing. **Sven F. Seys:** conceptualization, writing – original draft, writing – review and editing, funding acquisition, supervision, formal analysis, project administration. **Joost de Kinderen:** conceptualization, writing – review and editing, writing – original draft, supervision, formal analysis, project administration. **Valérie Hox:** conceptualization, investigation, writing – review and editing. **Carlo Cavaliere:** conceptualization, investigation, writing – review and editing. **Alexandros Andrianakis:** conceptualization, investigation, writing – review and editing. **Sven Schneider:** conceptualization, investigation, writing – review and editing. **Martin Wagenmann:** conceptualization, investigation, writing – review and editing. **Martin Bruch:** conceptualization, investigation, writing – review and editing. **Adriana Izquierdo‐Dominguez:** conceptualization, investigation, writing – review and editing. **Xavier Gonzalez‐Compta:** conceptualization, investigation, writing – review and editing. **Laura Pardo Muñoz:** conceptualization, investigation, writing – review and editing. **Laura Van Gerven:** conceptualization, investigation, writing – review and editing. **Peter W. Hellings:** conceptualization, investigation, writing – review and editing. **Geoffrey Mortuaire:** conceptualization, investigation, writing – review and editing. **Martin Burian:** investigation, resources, writing – review and editing. **Mireia Golet‐Fors:** investigation, writing – review and editing. **Mathilde Moyaert:** investigation, writing – review and editing. **Peter‐Valentin Tomazic:** conceptualization, investigation. **Giulia Bettio:** formal analysis, writing – review and editing. **Marco de Vincentiis:** investigation, writing – review and editing. **Zuzana Diamant:** conceptualization, writing – review and editing, supervision. **Julia Eckl‐Dorna:** conceptualization, investigation, writing – review and editing. **Gert Mariën:** conceptualization, funding acquisition, supervision, project administration, writing – review and editing. **Simonetta Masieri:** investigation, writing – review and editing. **Christina Morgenstern:** formal analysis, writing – review and editing. **Kathrin Scheckenbach:** investigation, formal analysis, writing – review and editing. **Aldine Tu:** formal analysis, writing – review and editing. **Camilo Rodriguez van Strahlen:** investigation, writing – original draft, writing – review and editing, formal analysis. **Claus Bachert:** conceptualization, funding acquisition, writing – review and editing, supervision.

## Ethics Statement

The study was approved by the local institutional review boards (France: not required as long as clear information and consent has been reported) and registered at clinicaltrials.gov (NCT04670172).

## Conflicts of Interest

C.B.: Claus Bachert reports grants or contracts from GSK, Sanofi, Novartis, Galenus Health. G.B.: Giulia Bettio is an employee of Galenus Health. Z.D.: Zuzana Diamant reports consulting fees and/or payment for lectures in the past 3 years: from Arcede, Biosion, Foresee Pharmaceuticals, Galenus Health, GlaxoSmithKline, Hippo‐Dx, Pleuran, Sanofi‐Genzyme and QPS‐NL. Leadership role in EUFOREA (asthma expert panel chair 2020–2023), associate editorships at Springer (RESNI/MedNet 2011–2024), Respiratory Medicine (2004‐ongoing) and Allergy (2018–2024). J.E.D.: Julia Eckl‐Dorna reports grants (institution) from Astra Zeneca, Novartis, payment for lectures from Allergopharma, participation on advisory board from GSK, Astra Zeneca, Bencard. V.H.: Valérie Hox reports payments for lectures from Novartis, participation to advisory boards of Sanofi, GSK and leadership role in EAACI. J.K.: Joost de Kinderen is a partner and shareholder of Galenus Health. G.M.: Gert Mariën is a partner and shareholder of Galenus Health. S.M.: Simonetta Masieri reports consulting fees from GSK, Sanofi, Novartis, Astra Zeneca, support for attending meetings from LoFarma, Sanofi, participation on advisory board from AstraZeneca, Sanofi, Novartis. S.F.S.: Sven Seys is an employee of Galenus Health. S.S.: Sven Schneider reports grants, payment for lectures and participation on advisory board from Sanofi, Astra Zeneca and GSK. P.V.T.: Peter‐Valentin Tomazic: No disclosures related to this manuscript. M.W.: Martin Wagenmann reports grants from ALK‐Abello, GSK, Regeneron, Astra Zeneca, Novartis, Sanofi, Takeda, consulting fees from ALK, Genzyme, Novartis, Stallergenes, Astra Zeneca, GSK, Sanofi, payment for lectures from ALK‐Abello, Astra Zeneca, GSK, Leti Pharma, Sanofi, Allergopharma, Bencard Allergie, Infectopharm, Novartis, Stallergenes, Leadership role in DGAKI (executive committee). A.I.‐D.: Adriana Izquierdo‐Domínguez reports payments for lectures from GlaxoSmithKline, Viatris, Sanofi, Loafarma, Novartis, Menarini, Organon, Uriach, Inmunotek, Leti, Diater, and AstraZeneca. AA: speaker fees from GSK and advisory board member for AstraZeneca. C.R.V.: Camilo Rodriguez van Strahlen reports receiving lectures fees from GSK and Proxymm Conferences. M.Br.: Martin Bruch reports grants, payment for lectures and participation on advisory boards from Sanofi, GSK, Astra Zeneca. MBu: No disclosures related to this manuscript. M.G.Z.: No disclosures related to this manuscript. L.P.M.: No disclosures related to this manuscript. M.G.F.: No disclosures related to this manuscript. C.M.: No disclosures related to this manuscript. K.S.: No disclosures related to this manuscript. A.T.: No disclosures related to this manuscript. Md.V.: No disclosures related to this manuscript.

## Supporting information


Supporting Information S1

## Data Availability

The authors have nothing to report.
