# Supplementary material for: Real‐World Effectiveness of Mepolizumab in Patients With Chronic Rhinosinusitis With Nasal Polyps: Findings From the European CRS Outcome Registry (CHRINOSOR)
Source: Clin Transl Allergy. 2026 Jan 31;16(2):e70153. doi: 10.1002/clt2.70153 (PMC12860572; doi:10.1002/clt2.70153)
Supplement: Supplementary file 1 — Supporting Information S1 [file CLT2-16-e70153-s001.docx]

**ONLINE REPOSITORY**

**List participating centers**

| COUNTRY | CITY | CENTER | NUMBER OF PATIENTS |
| --- | --- | --- | --- |
| Austria | Graz | Medizinisches Universitätzklinikum Graz | 3 |
|  | Vienna | Medizinisches Universitätzklinikum Wien | 3 |
|  | Linz | Ordensklinium Linz | 2 |
| Belgium | Leuven | University Hospitals Leuven | 4 |
|  | Brussels | Cliniques Universitaires Saint-Luc | 22 |
| France | Lille | Centre Hopitalier Universitaires de Lille | 6 |
| Germany | Dusseldorf | Universitätsklinikum Düsseldorf | 7 |
| Italy | Rome | Policlinico Umberto I | 20 |
| Spain | Barcelona | Hospital Clinic Barcelona | 18 |
|  |  | Bellvitge Hospital | 10 |
|  |  | Hospital Universitari Germans Trias i Pujol | 8 |
|  |  | Hospital Universitario de Terrassa | 7 |

**Methods**

*Inclusion and exclusion criteria*

Patients without available follow-up data at 24 or 52 weeks (either lost to follow-up or patients who had no follow-up visit(s) yet or in case data was not recorded), or when the date of available follow-up data was outside the window of 24 and 52 weeks $\pm$ 8 weeks, or patients treated with concurrent biologic treatment (for CRSwNP as primary indication or another type 2 inflammatory condition), were not included. Patients treated concurrently with another biologic were not included. Patients treated with another biologic prior to the mepolizumab treatment for CRSwNP, were included. Patients were allowed to continue their appropriate medical treatment.

*Data collection and outcome measures*

Demographics (age, sex, smoking status, body mass index (BMI)), disease history (number of endoscopic sinus surgery (ESS) procedures, use of courses of systemic corticosteroid (SCS) in the past year, prior biologic use targeting type 2 diseases), the presence of physician-diagnosed comorbidities (asthma, non-steroidal anti-inflammatory drug (NSAID) exacerbated respiratory disease (N-ERD), any type of allergic disease) were documented at baseline. The following clinical outcomes were assessed at baseline and at the follow-up visits: NPS (0-4 on every side, total score 0-8), disease-specific health-related questionnaires: SNOT-22 on a total score of 110 (from 0: no impact on QoL to 110: extreme impact on QoL), Asthma Control Test (ACT) on total score of 25 (25 = total control of asthma) and CRS-specific symptoms via VAS (from 0 mm “no bothersome symptoms” to 100 mm “extremely bothersome symptoms”), total sinus symptoms (TSS), loss of smell (LoS) and nasal blockage (NB), blood eosinophil counts (BEC) (cells/mm^3^), serum total IgE levels (IU/ml), and the use of SCS in the year prior to biologic initiation and during treatment.

Adverse events were not structurally assessed and reported.

*Sample size calculation*

Sample size calculations for NPS and SNOT-22 scores were based on historical data from a prior mepolizumab phase 3 trial (Han et al., Lancet Resp Med, 2021). Using a one-sided significance level of 0.025 and ≥80% power to detect treatment effects at 52 weeks versus baseline, a minimum of 71 patients (NPS; Cohen’s d = 0.47) and 50 patients (SNOT-22; Cohen’s d = 0.56) were required.  Analyses were performed in R (v4.4.1 (2024-06-14, R Foundation for Statistical Computing, Vienna, Austria) using the *pwr* (v1.3-0) library.

*EPOS-EUFOREA 2023 response analysis*

In addition to EUFOREA 2021 criteria we also analysed response criteria as defined by the updated EPOS-EUFOREA 2023 recommendations: no response (0/5), poor-moderate (1-3/5), good-excellent (4-5/5). We applied the following parameters and cutoffs: reduced NPS (change in NPS equal or larger than 1), reduced need for systemic corticosteroids (no systemic corticosteroid use), improved quality of life (change in SNOT-22 equal or larger than 8.9), improved sense of smell (change in VAS loss of smell equal or larger than 20mm), reduced impact of comorbidities (change in ACT equal or larger than 3).

*Remission analysis*

We analysed the proportion of patients that could be qualified as under remission at 52 weeks in line with the EUFOREA expert-based definition: remission is defined as a prolonged state of control, without bothersome symptoms reported by the patient for at least 12 months, without the need for either oral corticosteroids or endoscopic sinus surgery (ESS), and without endoscopic signs of active disease. In our analysis, no bothersome symptoms was defined as VAS nasal blockage, VAS loss of smell and VAS total sinus symptoms all equal or less than 50mm). No signs of active disease was defined as NPS equal or smaller than 2 points.

**Results**

*Analysis of mepolizumab responders according to EPOS-EUFOREA 2023*

At 52 weeks, 11.1% of patients showed no response, 46.7% showed a poor-moderate response and 42.2% showed a good-excellent response.

*Analysis of SNOT-22 MCID to identify mepolizumab responders*

Phillips et al. (Rhinology 2021) reported a minimal clinically importance difference of 12 points for SNOT-22 in non-surgical patients. We therefore also analysed how much patients reached this MCID for SNOT-22 at 24 and 52 weeks of mepolizumab treatment. We found respectively 66.3% and 78.3% of patients with a SNOT-22 change to baseline of 12 or larger.

*Effectiveness of mepolizumab in patients with and without prior ESS*

Patients without prior ESS (15.0% of subjects) did not show a significant improvement in NPS and SNOT-22 at 24 weeks as well as NPS at 52 weeks, but a significant improvement was observed for SNOT-22 at 52 weeks (Figure S5 A, B).

Patients with prior ESS showed a significant improvement in both NPS and SNOT-22 at 24 weeks and at 52 weeks (Figure S5 A, B). Further stratification of these patients on the number of prior ESS showed that NPS improved only in patients with 1 and >3 procedures, but not in those with 2 and 3 procedures, whereas significant improvements in SNOT-22 were found in all ESS subgroups (Figure S5 C, D).

*Analysis of disease remission*

Remission could be assessed in 54 patients and 25.9% (14/54) fullfilled remission criteria at 52 weeks of treatment.

**Tables**

| **Country** | **Germany** | **Belgium** | **Austria** | **Spain** | **Italy** | **France** |
| --- | --- | --- | --- | --- | --- | --- |
| Centres (cities) | Düsseldorf | Brussels, Leuven | Graz, Linz, Vienna | Barcelona (4 centres) | Rome | Lille |
| **Mandatory criteria (all need to be met)** | - ≥18 years - CRSwNP (confirmation by objective methods) - Severe - Inadequate control with continuous INCS - Systemic corticosteroids, and/or previous ESS | - ≥18 years - Bilateral polyps confirmed by endoscopy; descriptively, schematically, or photographically documented - Inadequate symptom control with INCS - previous ESS (unless contraindicated) | - ≥18 years - CRSwNP - Severe - Inadequate symptom control with INCS | - ≥18 years - Bilateral CRSwNP - VAS TSS > 7-10 cm and/or SNOT-22 > 50 - previous ESS (unless contraindicated) | - ≥18 years - Diffuse CRSwNP confirmed by endoscopy and CT - Inadequate symptom control with INCS - At least two cycles of systemic corticosteroid over the last year and/or - previous ESS | - ≥18 years - Bilateral polyps - ESS in last 3 years (or contraindicated) - Nasal steroids & cleaning nose with salty water regularly for≥3 months (or contraindicated) - Type-2 inflammation, defined by EOS in polyp tissue, eosinophilia in the blood or high Total IgE |
| **Plus optional criteria (some need to be met)** |  | **Needs to meet at least 3 out of 4 numbered criteria:** | **Needs to meet at least 1 out of 2 numbered criteria:** | **Needs to meet at least 1 out of 4 numbered criteria:** | **Needs to meet at least 1 out of 2 numbered criteria:** | **Needs to meet at least 3 out of 5 numbered criteria:** |
|  |  | 1. At least 2 cycle of SCS in last 2 years (unless contraindicated) 2. Anosmia 3. BEC ≥ cells/µL at start or any moment 12 months prior to start treatment, and/or Tissue eosinophilia confirmed by histologic analysis 4. Confirmed asthma diagnosis | 1. Systemic corticosteroids, 2. previous ESS | 1. At least two cycles of SCS over the last year (or contraindication due to intolerance /allergy for SCS) 2. Anosmia or severe hyposmia, measured by VAS LoS >7 (0-10 scale), 3. Type 2 inflammation: measured by BEC ≥ 300 cells/µL, and/or Tissue eosinophils 10 cells/HPF, and/or Total serum IgE > 100 UI/m>. 4. ****Asthma: measured by need for Maintenance ICS | 1. NPS ≥ 5 2. SNOT-22 ≥ 50 | 1. SNOT-22 ≥ 50 2. NPS ≥2 each site & ≥5 total 3. Need for systemic steroid at least 2 courses on low dose treatments during 3 months 4. Anosmia 5. Asthma defined by the need for maintenance ICS |

Hard criteria (method, metric, and cut-off value / dose / frequency value defined)

Semi-hard criteria (method and metric, but no cut-off value / dose / frequency defined)

Semi-soft criteria (optional method and metric, but no cut-off value / dose / frequency defined)

Soft criteria (no method, metric, or cut-off value / dose / frequency defined)

**Table S1. National reimbursement criteria for mepolizumab treatment in CRSwNP patients.**

| **Stratification by** | **Sub-groups** | | **# patients** | **Composite responder criteria** | **% responders at 24 weeks** | | | | | | **% responders at 52 weeks** | | | | | **Difference from 24 to 52 weeks** | |  |
| --- | --- | --- | --- | --- | --- | --- | --- | --- | --- | --- | --- | --- | --- | --- | --- | --- | --- | --- |
| **Prior biologic** |  | Yes | 13 | 24w Criteria - at least 1 out of 5 criteria to be met | 11 | out of | 13 | = | 84,6% | 6 | | out of | 10 | = | 60,0% | | -24,6% | |
|  |  |  |  | 52w Criteria - all 4 out of 4 criteria to be met | - | out of | 11 | = | 0,0% | 2 | | out of | 9 | = | 22,2% | | +22,2% | |
|  |  | No | 97 | 24w Criteria - at least 1 out of 5 criteria to be met | 78 | out of | 91 | = | 85,7% | 64 | | out of | 79 | = | 81,0% | | -4,7% | |
|  |  |  |  | 52w Criteria - all 4 out of 4 criteria to be met | 13 | out of | 60 | = | 21,7% | 23 | | out of | 47 | = | 48,9% | | +27,3% | |
| **Discontinuation of Mepolizumab** |  | Yes | 19 | 24w Criteria - at least 1 out of 5 criteria to be met | 14 | out of | 19 | = | 73,7% | 5 | | out of | 6 | = | 83,3% | | +9,6% | |
|  |  |  |  | 52w Criteria - all 4 out of 4 criteria to be met | 2 | out of | 13 | = | 15,4% | 0 | | out of | 2 | = | 0,0% | | -15,4% | |
|  |  | No | 91 | 24w Criteria - at least 1 out of 5 criteria to be met | 75 | out of | 85 | = | 88,2% | 65 | | out of | 83 | = | 78,3% | | -9,9% | |
|  |  |  |  | 52w Criteria - all 4 out of 4 criteria to be met | 11 | out of | 58 | = | 19,0% | 25 | | out of | 54 | = | 46,3% | | +27,3% | |

**Table S2. Mepolizumab response stratified by baseline BEC, prior biologics and discontinuation of mepolizumab treatment.**

|  |  | **CHRINOSOR real-world study** | | | | **SYNAPSE phase 3 clinical trial** | | |
| --- | --- | --- | --- | --- | --- | --- | --- | --- |
|  |  | **(n=110)** | | | | **(n=206)** | | |
| Timepoint | Parameter | n | % | Median | Mean | % | Median | Mean |
| Baseline | Asthma | 110 | 86.4 |  |  | 68.0 |  |  |
|  | N-ERD | 94 | 36.2 |  |  | 21.8 |  |  |
|  | Prior ESS | 107 | 85.0 |  |  | 100.0 |  |  |
|  | Serum total IgE | 87 |  | 110.0 | 298.4 |  |  |  |
|  | Blood Eosinophil Count | 98 |  | 500.0 | 558.1 |  |  | 390.0 |
|  | SCS use past year | 108 | 70.4 |  |  | 51.5 |  |  |
|  | NPS | 107 |  | 5.0 | 4.4 |  | 5.0 | 5.4 |
|  | SNOT-22 | 98 |  | 56.0 | 57.0 |  | 64.0 | 63.7 |
|  | VAS TSS | 81 |  | 51.0 | 53.5 |  | 91.0 | 90.0 |
|  | VAS NB | 75 |  | 70.0 | 68.0 |  | 90.0 | 89.0 |
|  | VAS LoS | 83 |  | 97.0 | 83.6 |  | 100.0 | 91.0 |
| Timepoint | Parameter | n | % | Median | Mean | % | Median | Mean |
| 52 weeks | NPS | 88 |  | 3.0 | 2.7 |  | 4.0 | 4.5 |
|  | SNOT-22 | 73 |  | 29.0 | 30.2 |  | 34.0 | 34.3 |
|  | VAS TSS | 71 |  | 28.5 | 35.3 |  | 46.2 | 47.0 |
|  | VAS NB | 66 |  | 30.0 | 36.2 |  | 45.9 | 47.0 |
|  | VAS LoS | 77 |  | 52.5 | 54.1 |  | 47.0 | 63.0 |
|  | SCS use | 65 | 30.8 |  |  | 25.0 |  |  |
| Endpoint outcomes | Parameter | n | % | Median | Mean | % | Median | Mean |
| Co-primary endpoint | reduction in NPS | 88 |  | -2.0 | -1.7 |  | -1.0 | -0.9 |
| Co-primary endpoint | reduction in VAS NB | 66 |  | -40.0 | -31.8 |  | -44.1 | -42.0 |
| Secondary endpoint | reduction in SNOT-22 | 73 |  | -27.0 | -26.8 |  | -30.0 | -29.4 |
| Secondary endpoint | reduction in VAS TSS | 71 |  | -22.5 | -18.1 |  | -44.8 | -43.0 |
| Secondary endpoint | reduction in VAS LoS | 77 |  | -44.5 | -29.4 |  | -53.0 | -28.0 |
| Secondary endpoint | reduction in use SCS | 65 | 39.6 |  |  | 26.5 |  |  |
|  | % patients with NPS improvement >=2 | 39 | 47.6 |  |  | 36.0 |  |  |
|  | % patients with NPS improvement >=1 | 49 | 59.8 |  |  | 50.6 |  |  |

**Table S3. Comparison of mepolizumab effectiveness in CHRINOSOR versus the SYNAPSE study results**

**Figures**

**
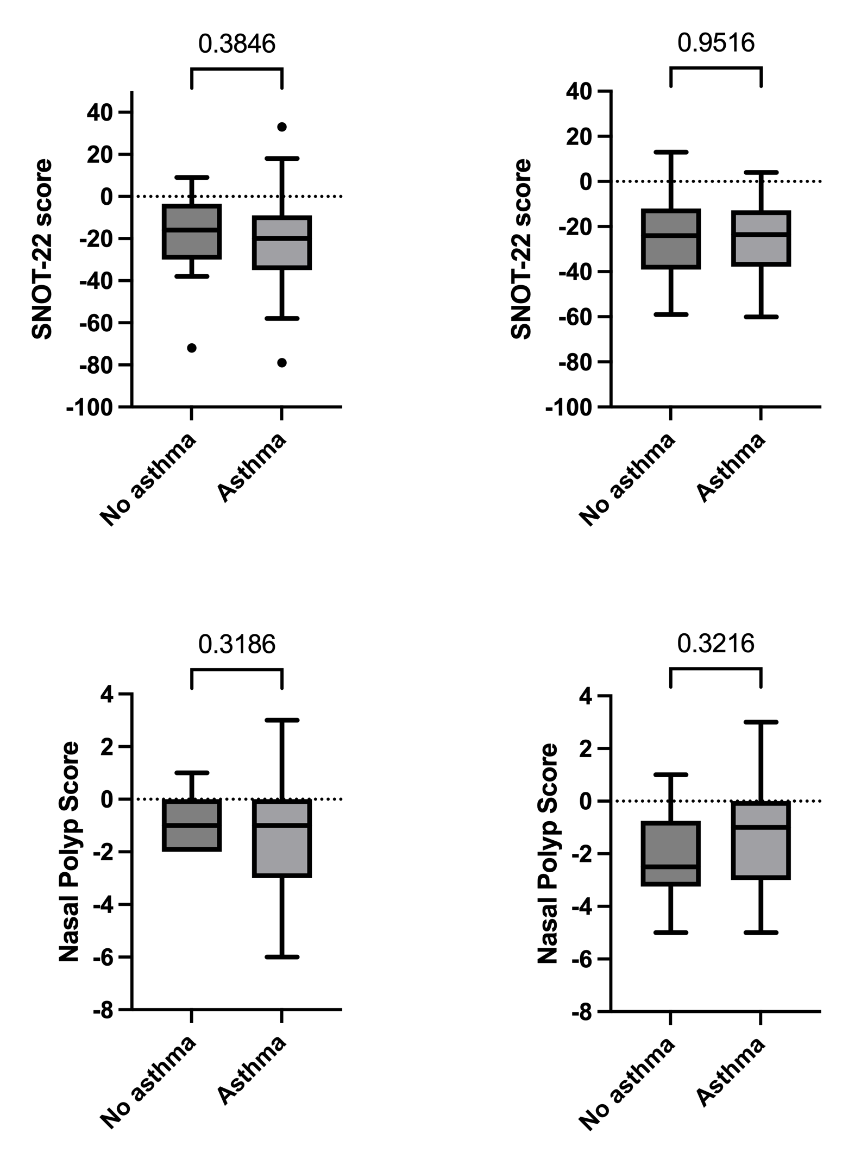
**

**Figure S1. SNOT-22 and NPS change to baseline at 24 weeks (left) and 52 weeks (right) stratified by the presence of comorbid asthma.**

Data are presented as Tukey box-and-whisker plots. Between-group comparison was performed by Mann-Whitney test.

**
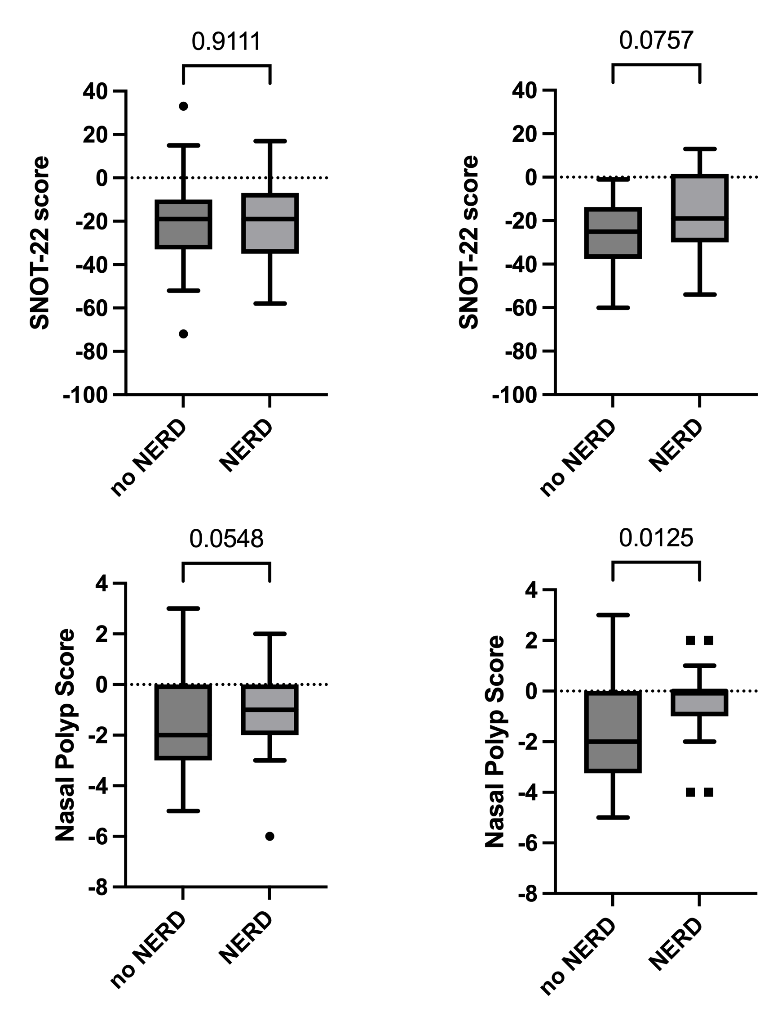
**

**Figure S2. SNOT-22 and NPS change to baseline at 24 weeks (left) and 52 weeks (right) stratified by the presence of N-ERD.**

Data are presented as Tukey box-and-whisker plots. Between-group comparison was performed by Mann-Whitney test.

**
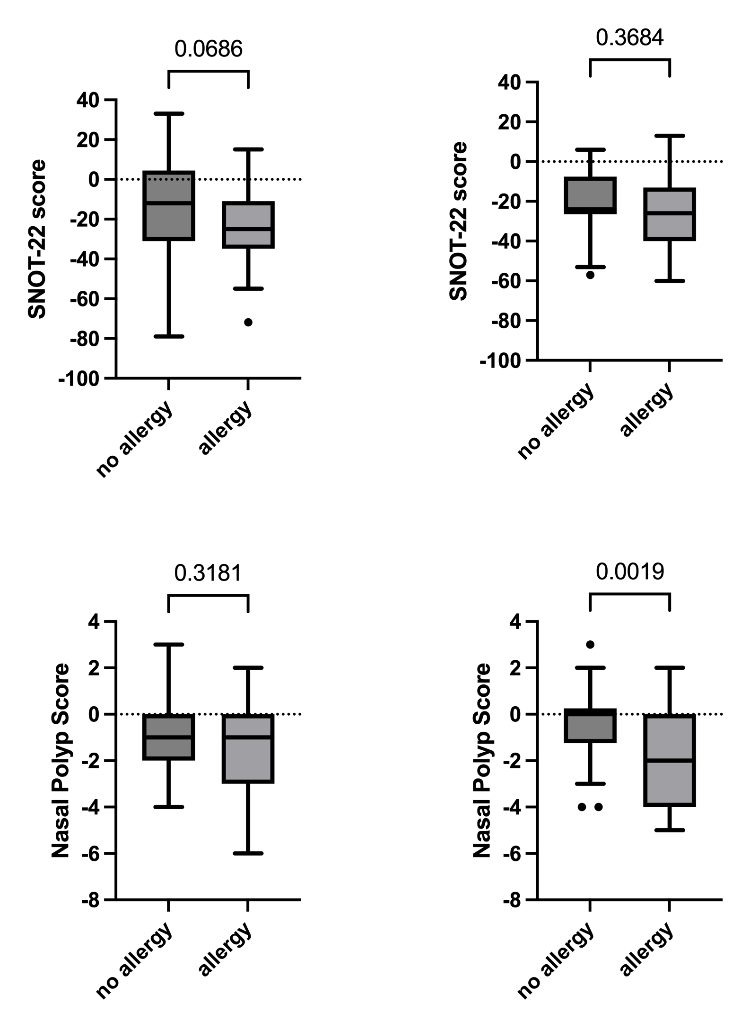
**

**Figure S3. Figure S2. SNOT-22 and NPS change to baseline at 24 weeks (left) and 52 weeks (right) stratified by the presence of allergy.**

Data are presented as Tukey box-and-whisker plots. Between-group comparison was performed by Mann-Whitney test.

**
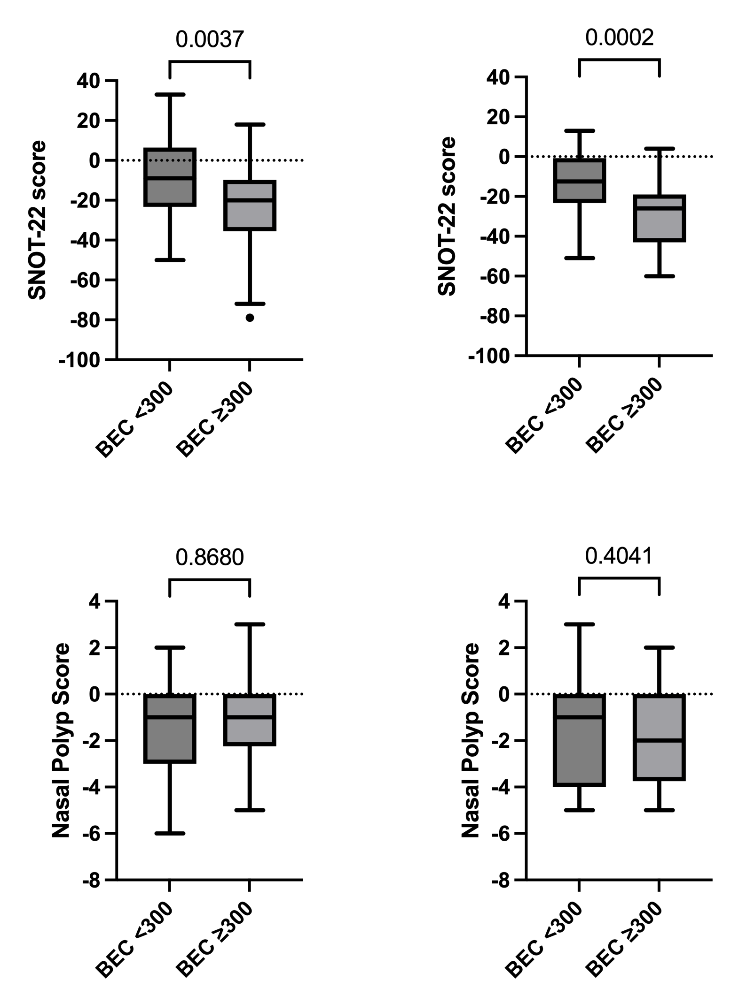
**

**Figure S4. Figure S3. Figure S2. SNOT-22 and NPS change to baseline at 24 weeks (left) and 52 weeks (right) stratified by the presence of blood eosinophil counts.**

Data are presented as Tukey box-and-whisker plots. Between-group comparison was performed by Mann-Whitney test.

**
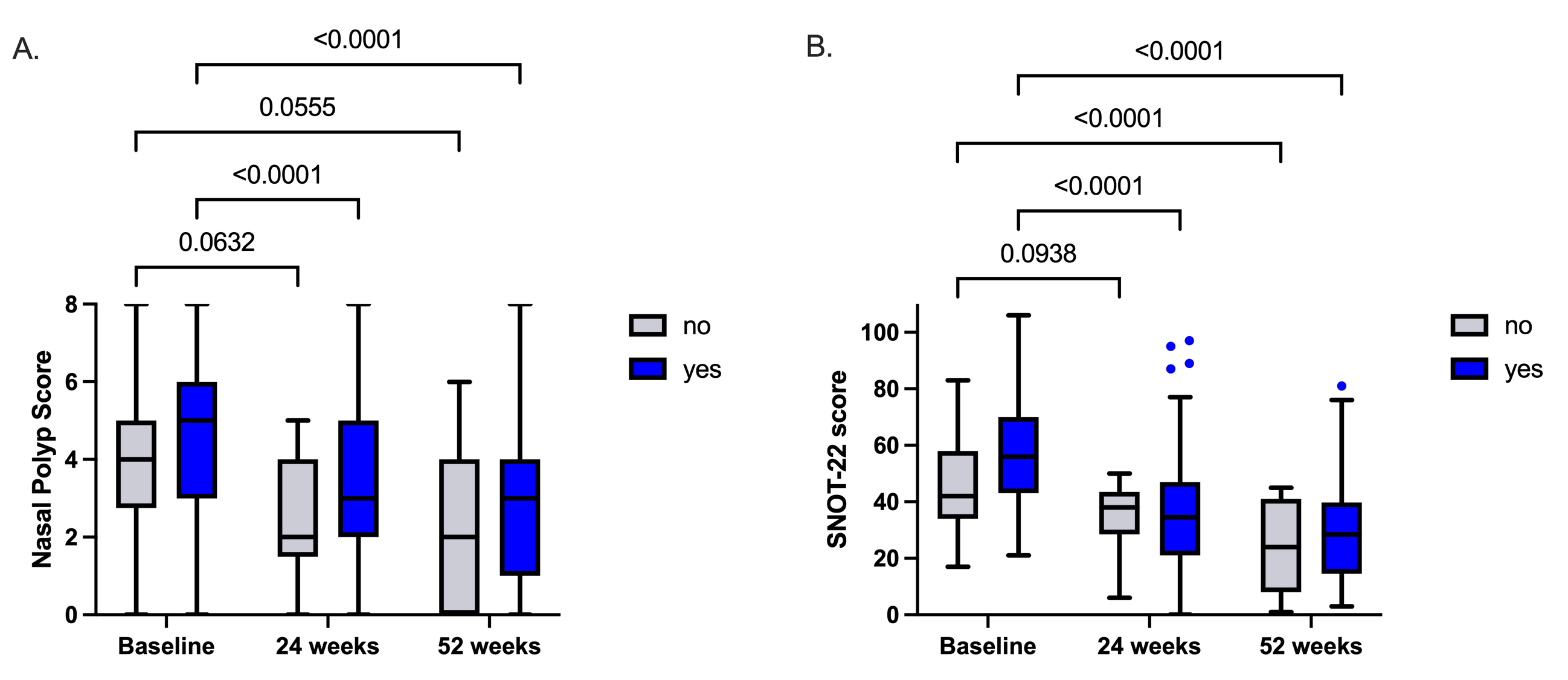
**

**
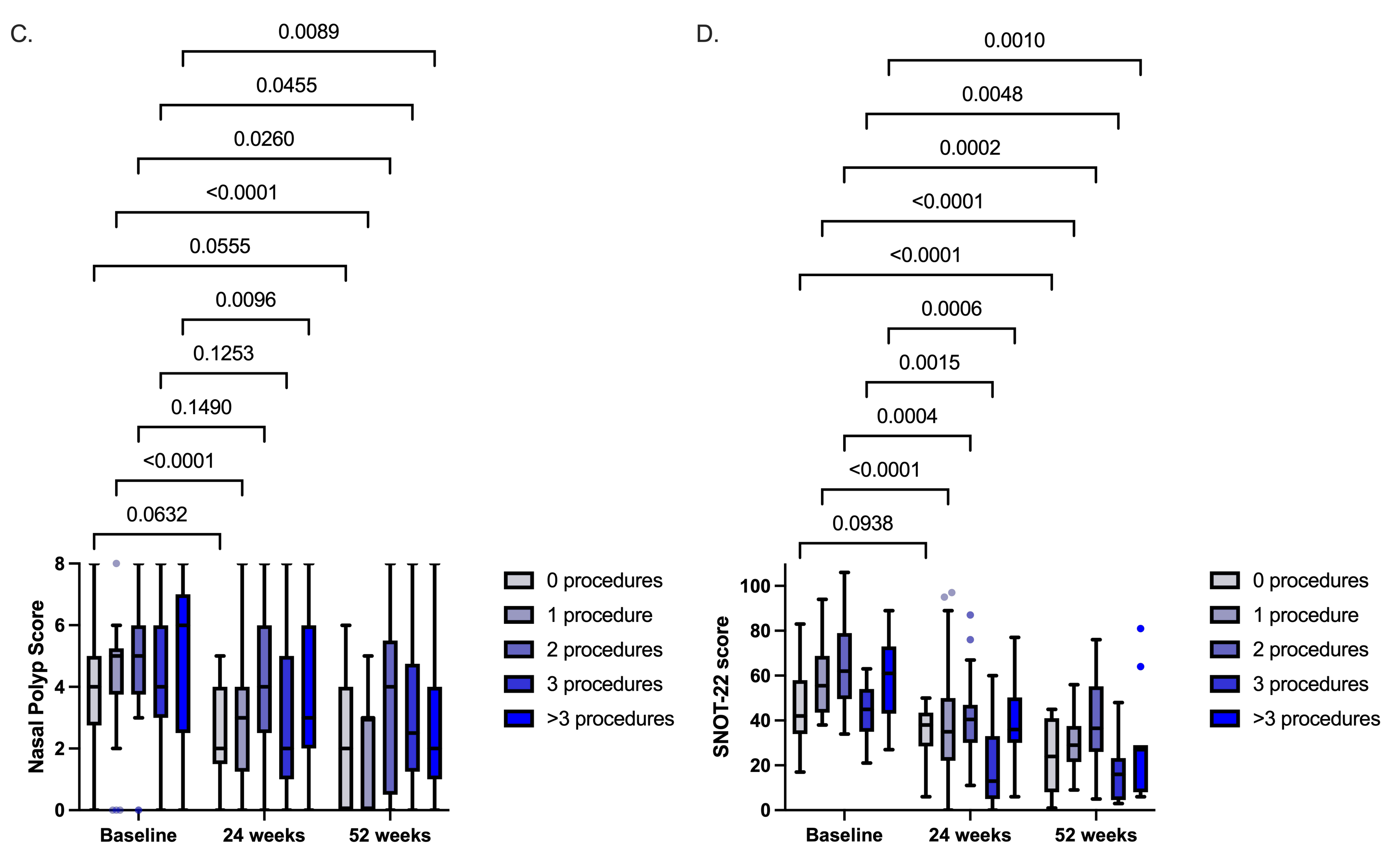
**

**Figure S5. Mepolizumab treatment effect on NPS and SNOT-22, stratified by the number of prior ESS.**

A, Patient numbers of NPS: ESS 0: 14, 9 and 13; ESS 1: 34, 32 and 27; ESS 2: 22, 21 and 17; ESS 3: 15, 13 and 12; ESS $\geq$3: 17, 16 and 13. B, Patient numbers of SNOT-22: ESS 0: 11, 9 and 7; ESS 1: 32, 31 and 25; ESS 2: 21, 20 and 16; ESS 3: 15, 15 and 10; ESS $\geq$3: 15, 16 and 11. Data are presented as Tukey box-and-whisker plots. Within-group comparison was performed by mixed effects model and Dunnett multiple testing comparison.
